# Supplementary material for: Benchmarking and integrating human B-cell receptor genomic and antibody proteomic profiling
Source: NPJ Syst Biol Appl. 2024 Jul 12;10:73. doi: 10.1038/s41540-024-00402-z (PMC11245537; doi:10.1038/s41540-024-00402-z)
Supplement: Supplementary file 1 — Supplementary materials [file 41540_2024_402_MOESM1_ESM.pdf]

Supplementary materials

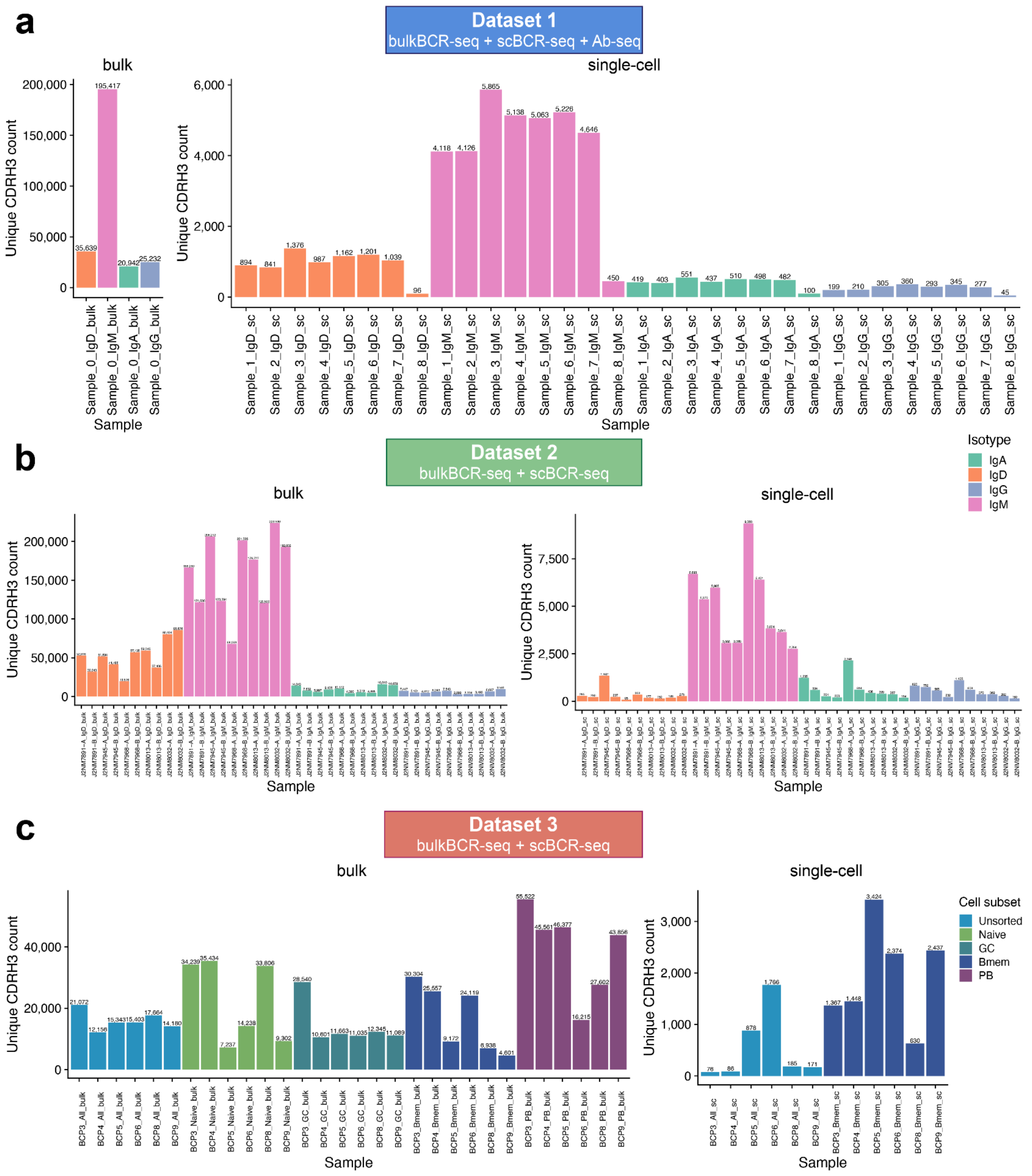

Supplementary figure 1: CDRH3 counts are mostly consistent across samples of the same isotype and B-cell subset. Each bar represents the number of unique CDRH3 sequences in each sequencing sample from **(a)** Dataset 1, **(b)** Dataset 2, and **(c)** Dataset 3 (Unsorted: all B cells, Naive: naive B cells, GC: germinal center B cells, Bmem:

memory B cells, PB: plasmablast). Samples were separated into bulkBCR-seq and scBCR-seq samples, colored by isotype (Dataset 1 and Dataset 2) or B-cell subset (Dataset 3). Relates to Figure 2.

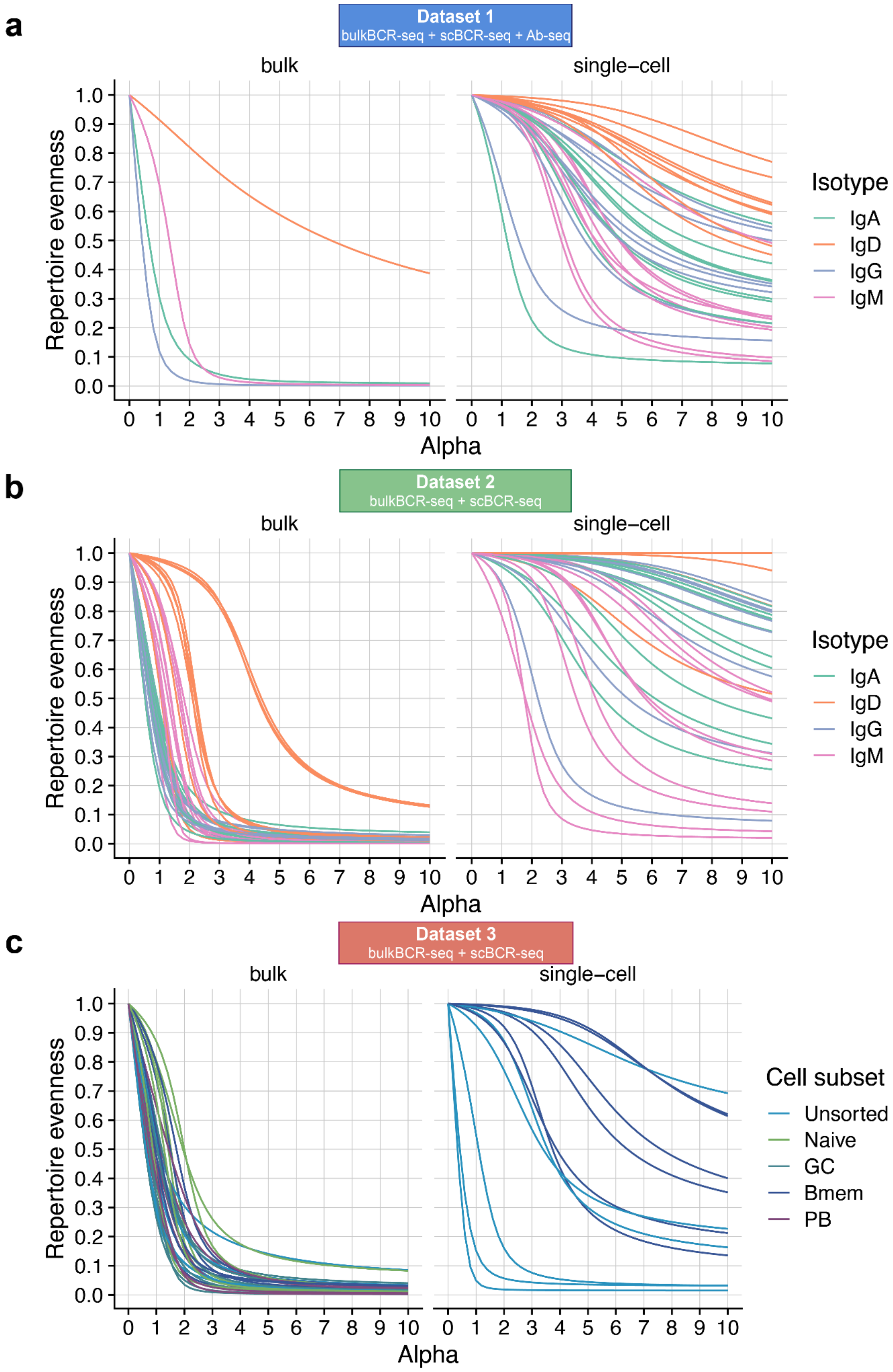

Supplementary figure 2: Clonal expansion is less prominent in IgD and naive B cell samples. Each line represents the repertoire evenness profile (see Methods) of each sample from **(a)** Dataset 1, **(b)** Dataset 2, and **(c)** Dataset 3 (Unsorted: all B cells, Naive: naive B cells, GC: germinal center B cells, Bmem: memory B cells, PB: plasmablast). Samples were separated into bulkBCR-seq and scBCR-seq samples, colored by isotype (Dataset 1 and Dataset 2) or B-cell subset (Dataset 3). Relates to Figure 2.

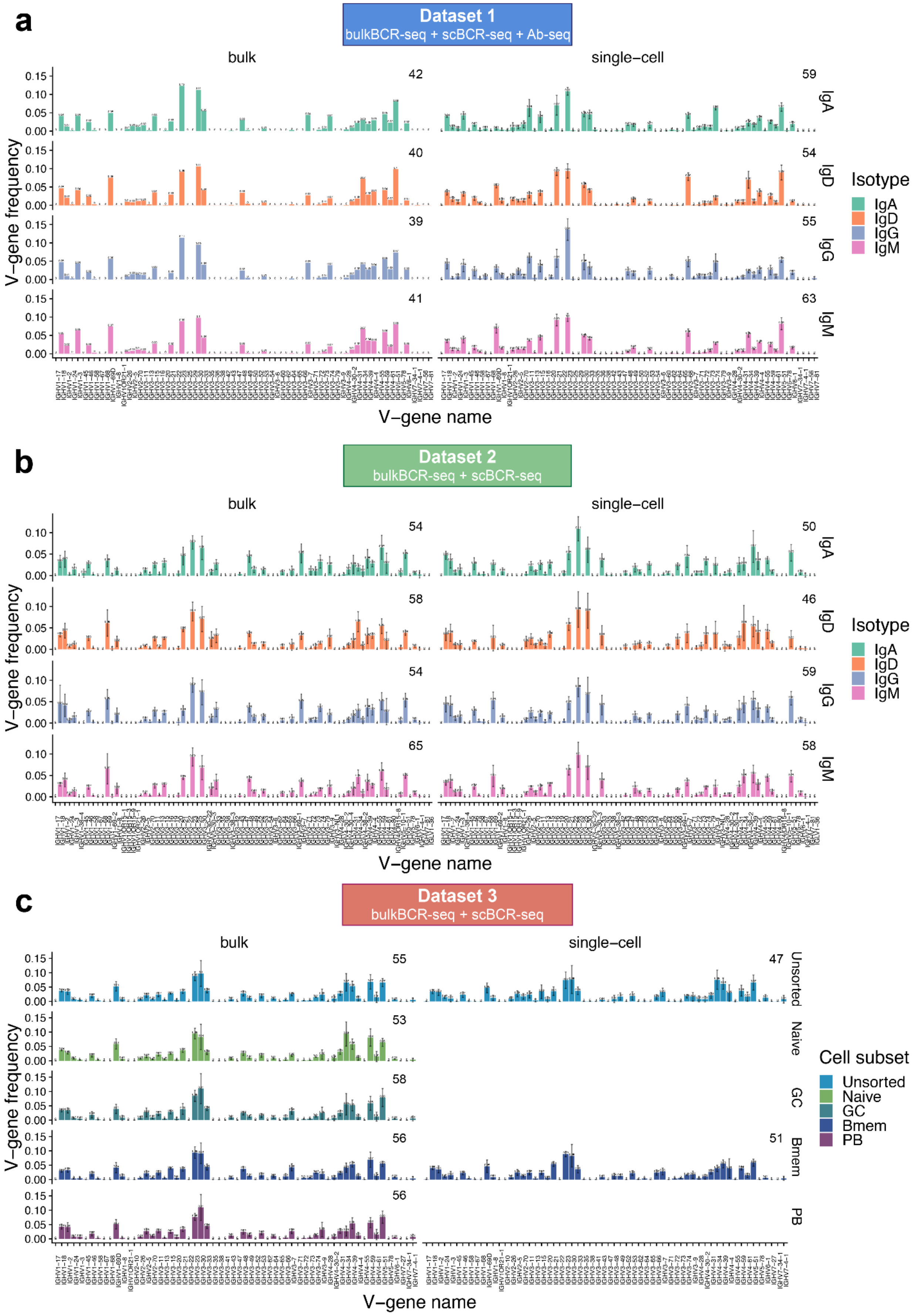
a
Dataset 1
bulkBCR-seq + scBCR-seq + Ab-seq
bulk
single-cell
V-gene frequency
0.15
0.10
0.05
0.00
42
59
40
54
39
55
41
63
IgA
IgD
IgG
IgM
V-gene name
Isotype
IgA
IgD
IgG
IgM
b
Dataset 2
bulkBCR-seq + scBCR-seq
bulk
single-cell
V-gene frequency
0.10
0.05
0.00
54
50
58
46
54
59
65
58
IgA
IgD
IgG
IgM
V-gene name
Isotype
IgA
IgD
IgG
IgM
c
Dataset 3
bulkBCR-seq + scBCR-seq
bulk
single-cell
V-gene frequency
0.15
0.10
0.05
0.00
55
47
53
58
56
51
56
Unsorted
Naive
GC
Bmem
PB
V-gene name
Cell subset
Unsorted
Naive
GC
Bmem
PB

Supplementary figure 3: VH-gene usage frequency is similar between sample groups. Each bar represents the mean VH gene frequency, and each error bar represents the standard deviation of the mean VH gene in proportion to all VH genes in a sample group, defined by an isotype in **(a)** Dataset 1 and **(b)** Dataset 2, or by a B-cell subset (Unsorted: all B cells, Naive: naive B cells, GC: germinal center B cells, Bmem: memory B cells, PB: plasmablast) in **(c)** Dataset 3. The data was presented as mean + standard deviation. Numbers in the top right corner annotate the number of unique VH genes in each sample group. Relates to Figure 2.

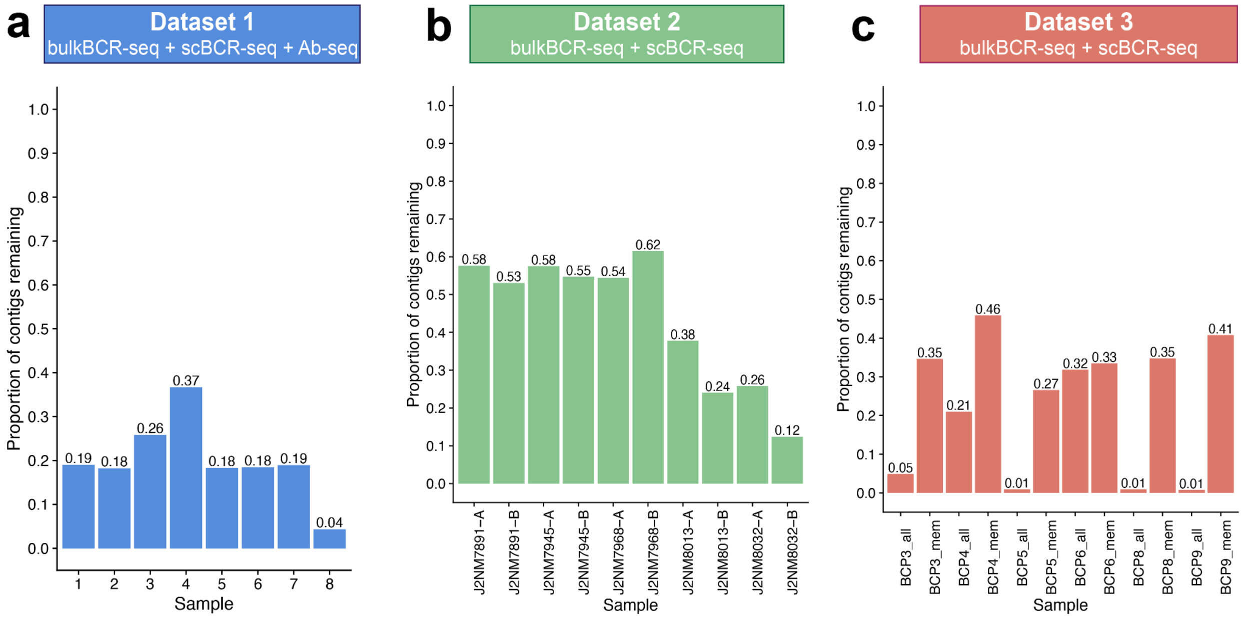

Supplementary figure 4: Single-cell contig counts after pre-processing varied between datasets. Each bar represents the proportion of single-cell contigs remaining after scBCR-seq data (see Methods) in **(a)** Dataset 1, **(b)** Dataset 2, and **(c)** Dataset 3 underwent quality control selection, including the presence of CDR3 sequence and identified isotype, actual single-cell droplets, correct pairing between heavy and light chain. Relates to Figure 2.

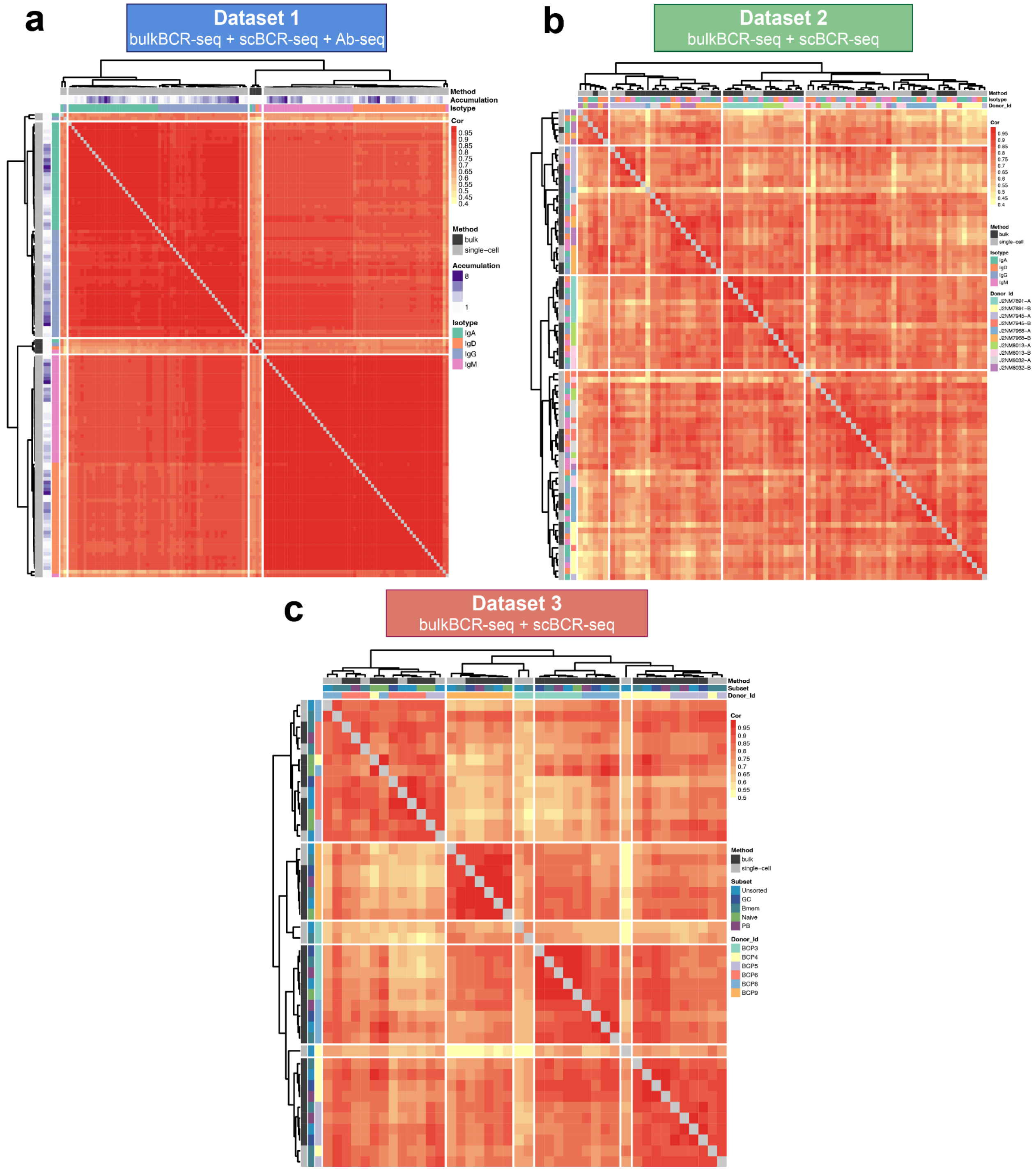

Supplementary figure 5: High VH-gene usage Pearson correlation overall between samples. Each cell represents the Pearson correlation of all VH-gene frequencies between two samples, colored by correlation value. Pairwise comparisons were clustered by Ward's method. Rows and columns were annotated by sequencing method, isotype (Dataset 1 and Dataset 2), number of scBCR-seq replicates merged (Dataset 1), and B-cell subset (Unsorted: all B cells, Naive: naive B cells, GC: germinal center B cells, Bmem: memory B cells, PB: plasmablast) (Dataset 3). Relates to Figure 3.

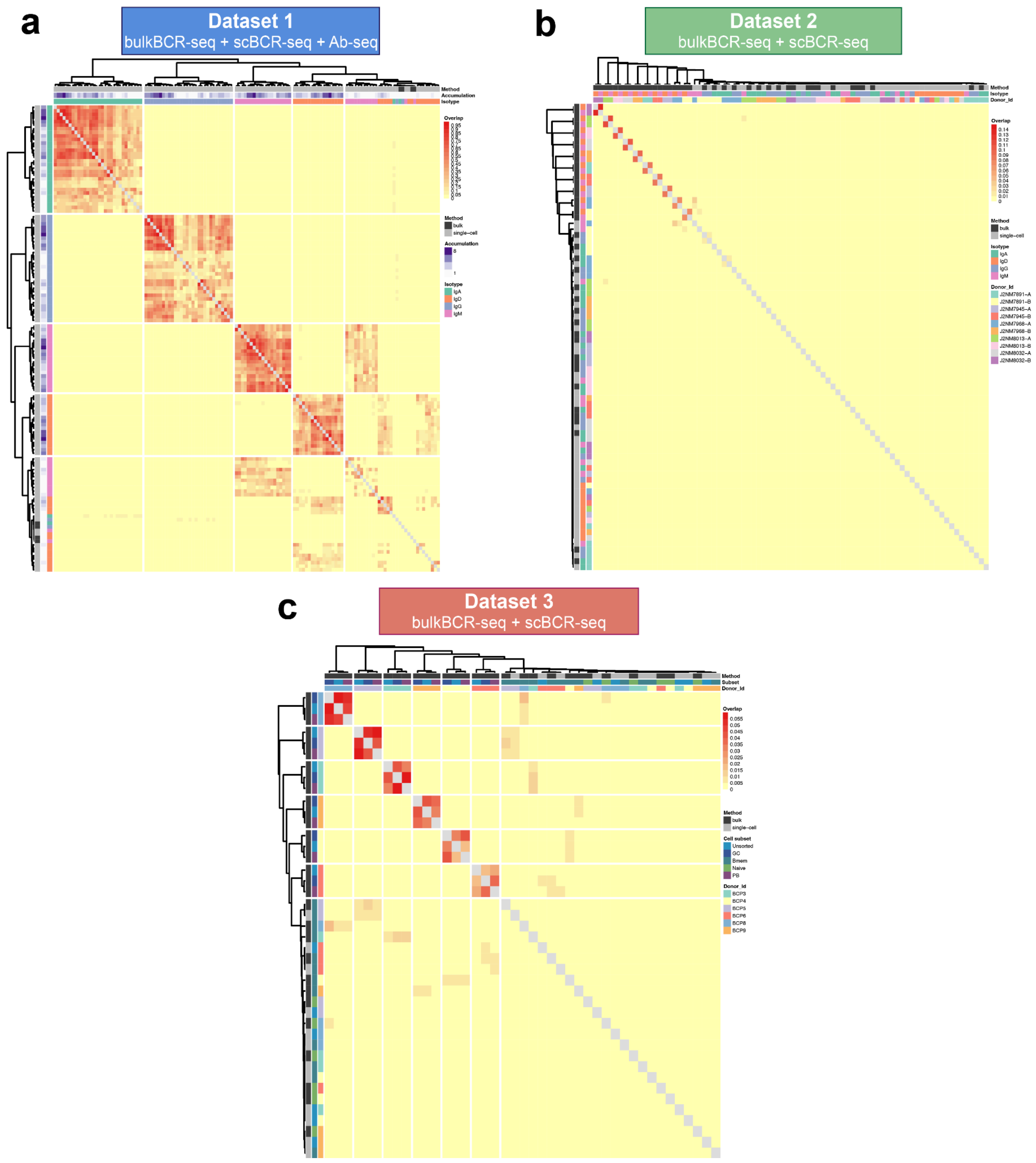

Supplementary figure 6: **Low CDRH3 Jaccard overlap overall between samples.** Each cell represents the Jaccard overlap index of shared CDRH3 sequences between two samples, colored by overlap value. Pairwise comparisons were clustered by Ward's method. Rows and columns were annotated by sequencing method, isotype (Dataset 1 and Dataset 2), number of scBCR-seq replicates merged (Dataset 1), and B-cell subset (Unsorted: all B cells, Naive: naive B cells, GC: germinal center B cells, Bmem: memory B cells, PB: plasmablast) (Dataset 3). Relates to Figure 4.

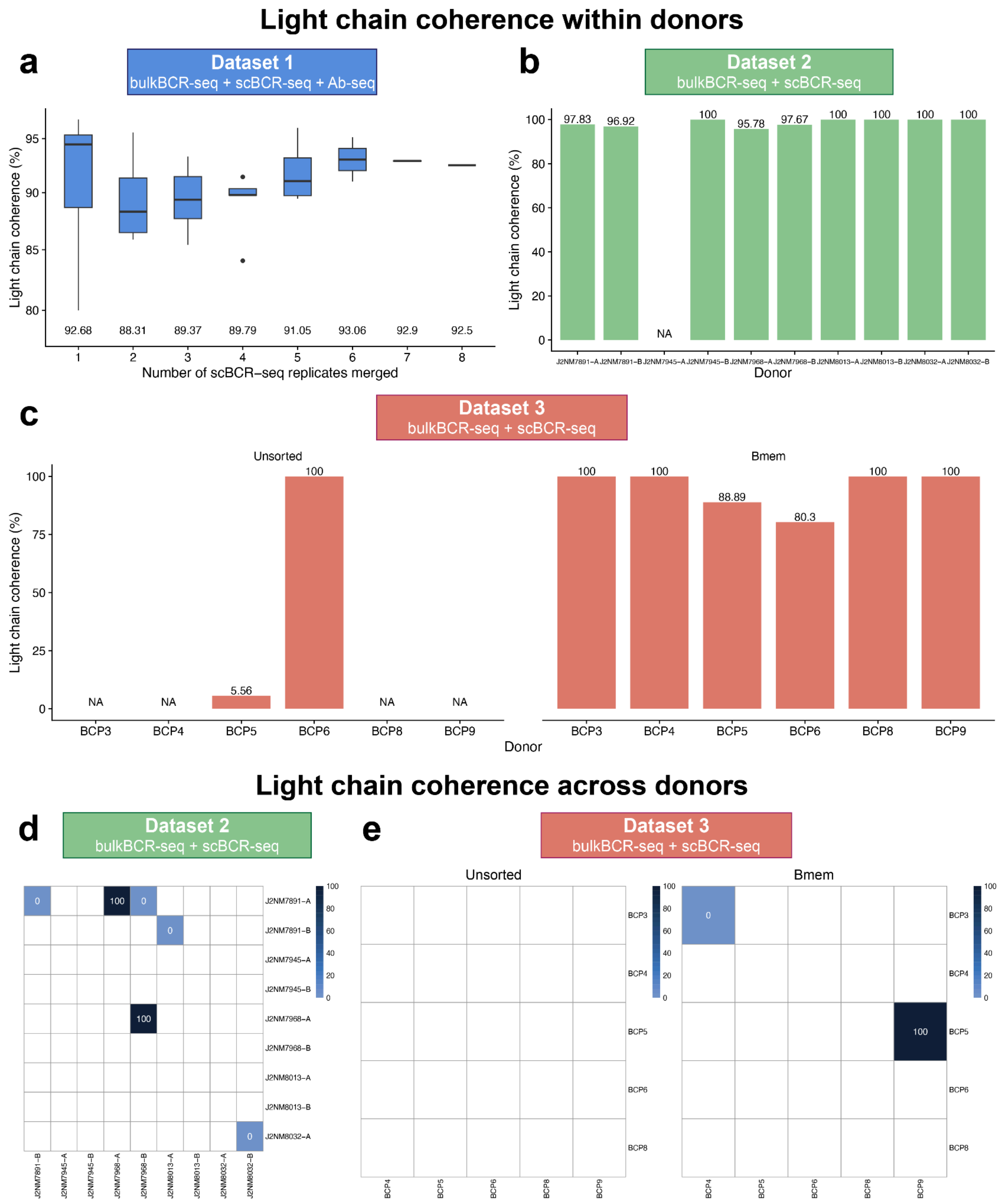

Supplementary figure 7: Light chain coherence values are unchanged with increasing replicates and higher in memory B cells than unsorted B cells. Light chain coherence within and across individuals (see Methods) were calculated for the scBCR-seq data of all three datasets. **(a)** Light chain coherence within an individual for Dataset 1 in regard to the number of scBCR-seq replicates merged together. Median coherence values are displayed under each boxplot. **(b)** Light chain coherence within individuals for Dataset 2 by donor. **(c)** Light chain coherence within

individuals for Dataset 3 by donor, separated by B-cell subset (unsorted B cells or memory B cells). **(d)** Pairwise light chain coherence across individuals for Dataset 2. **(e)** Pairwise light chain coherence across individuals for Dataset 3, separated by B-cell subset (unsorted B cells or memory B cells). Light chain coherence values were not calculated if there were no cell pairs meeting the criteria for light chain coherence evaluation.

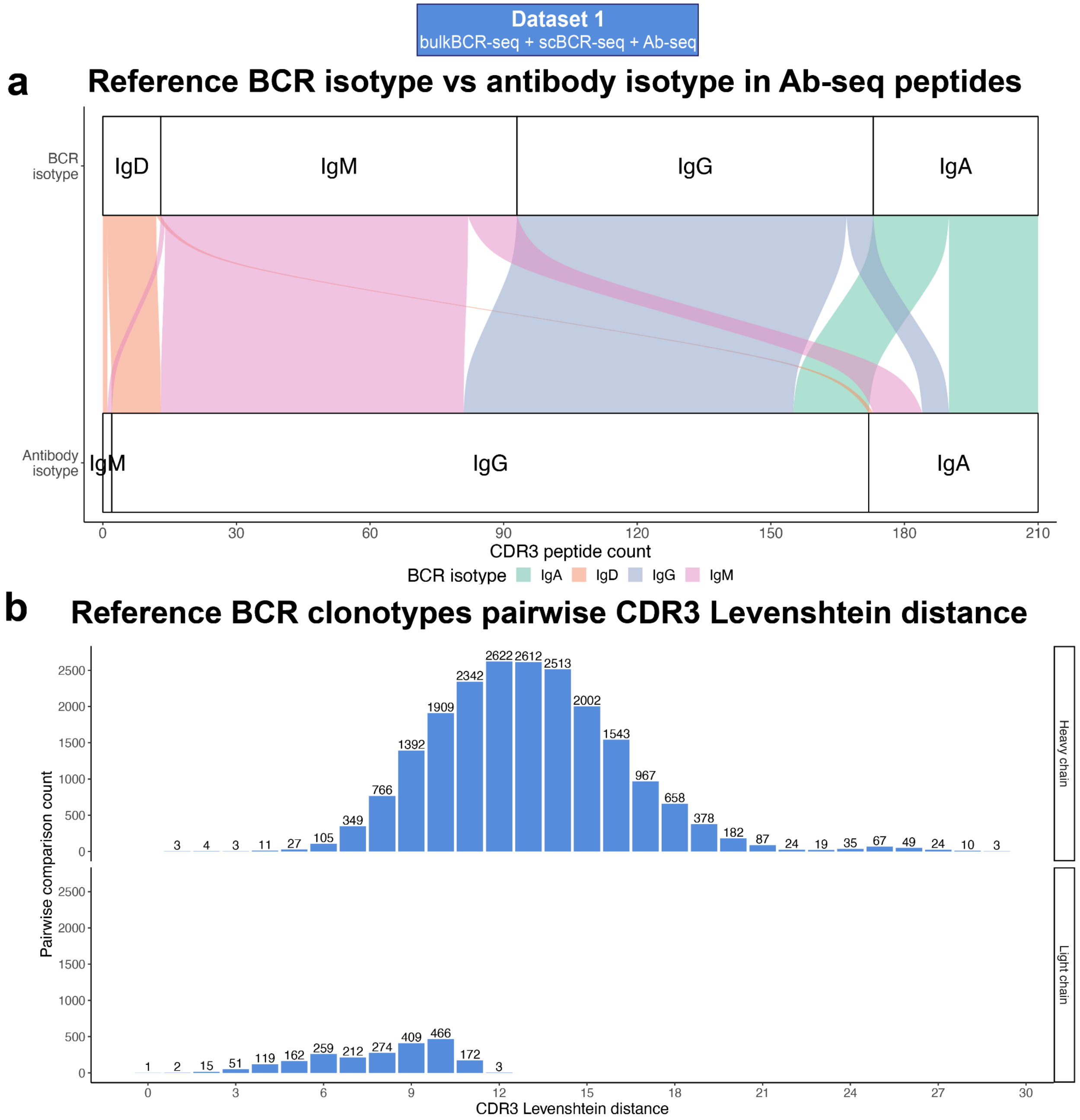

Supplementary figure 8: Most BCR clonotypes detected by Ab-seq underwent class switching, and differed by CDR3 sequence. (a) Distribution of isotypes of Ab-seq peptides between reference BCRs and serum antibodies. From Ab-seq CDR3 peptides that uniquely mapped to only one reference by MaxQuant, the identified reference BCR clonotypes were examined for correspondence in isotype between BCR form and serum antibody form. **(b)** Levenshtein (edit) distance distribution between pairs of reference CDR3 amino acid sequences identified by Ab-seq peptides by VH and VL chain.

Supplementary table 1. Overview of the cDNA synthesis primers. Letters in bold indicated the UMI sequence, while letters underlined indicated overlap with Illumina Read2 sequence.

Primer name	Sequence
**3' primers**	
Hu_IgG	GGAGTTCAGACGTGTGCTCTTCCGATCT**HHHHHACAHHHHHACAHHHH**GCCAGGGGGAAGACCGATGGG
Hu_IgM	GGAGTTCAGACGTGTGCTCTTCCGATCT**HHHHHACAHHHHHACAHHHH**NHCCGACGGGGAATTCTCACAGGAGACGAGGGGGAAAAG
Hu_IgA	GGAGTTCAGACGTGTGCTCTTCCGATCT**HHHHHACAHHHHHACAHHHH**GAAGACCTTGGGGCTGGT
Hu_IgD	GGAGTTCAGACGTGTGCTCTTCCGATCT**HHHHHACAHHHHHACAHHHH**GGGTGTCTGCACCCTGATA
Hu_IgE	GGAGTTCAGACGTGTGCTCTTCCGATCT**HHHHHACAHHHHHACAHHHH**GAAGACGGATGGGCTCTGT
Hu_IgK	GGAGTTCAGACGTGTGCTCTTCCGATCT**HHHHHACAHHHHHACAHHHH**NGGGATAGAAGTTATTCAGCAGGCACACAACAGAG
Hu_IgL	GGAGTTCAGACGTGTGCTCTTCCGATCT**HHHHHACAHHHHHACAHHHH**TGGCTTGRAGCTCCTCAGAGGAGG

Supplementary table 2. Overview of the Multiplex PCR primers.

Primer name	Sequence
**3' primers**	
Read2U	GGAGTTCAGACGTGTGCTCTTCCGATCT
**5' primers**	
Hu_VH_MTPX_1	CCTACACGACGCTCTTCCGATCTGGTGGCAGCAGTCACAGATGCCTACTC
Hu_VH_MTPX_2	CCTACACGACGCTCTTCCGATCTGGTGGCAGCAGCCACAGGTGCCCACTC
Hu_VH_MTPX_3	CCTACACGACGCTCTTCCGATCTGGTGGCAGCAGCTACAGGTGTCCAGTC
Hu_VH_MTPX_4	CCTACACGACGCTCTTCCGATCTGGTGGSAGCAGCAACARGWGCCCACTC
Hu_VH_MTPX_5	CCTACACGACGCTCTTCCGATCTGCTGGCTGTAGCTCCAGGTGCTCACTC
Hu_VH_MTPX_6	CCTACACGACGCTCTTCCGATCTCCTGCTGCTGACCAYCCCTTCMTGGGTCTTGTC
Hu_VH_MTPX_7	CCTACACGACGCTCTTCCGATCTCCTGCTACTGACTGTCCCGTCCTGGGTCTTATC
Hu_VH_MTPX_8	CCTACACGACGCTCTTCCGATCTGGGTTTTCCTCGTTGCTCTTTTAAGAGGTGTCCAGTG
Hu_VH_MTPX_9	CCTACACGACGCTCTTCCGATCTGGGTTTTCCTTGTTGCTATTTTAAAAGGTGTCCARTG
Hu_VH_MTPX_10	CCTACACGACGCTCTTCCGATCTGGATTTTCCTTGCTGCTATTTTAAAAGGTGTCCAGTG
Hu_VH_MTPX_11	CCTACACGACGCTCTTCCGATCTGGGTTTTCCTTKTKGCTATWTTAGAAGGTGTCCAGTG
Hu_VH_MTPX_12	CCTACACGACGCTCTTCCGATCTGGTGGCRGCTCCCAGATGGGTCCTGTC
Hu_VH_MTPX_13	CCTACACGACGCTCTTCCGATCTCTGGCTGTTCTCCAAGGAGTCTGTG
Hu_VH_MTPX_14	CCTACACGACGCTCTTCCGATCTGGCCTCCCATGGGGTGTCCTGTC
Hu_VH_MTPX_15	CCTACACGACGCTCTTCCGATCTGGTGGCAGCAGCAACAGGTGCCCACT
Hu_VH_MTPX_16	CACTCTTTCCCTACACGACGCTCTTCCGATCTATGGAACTGGGGCTCCGCTGGGTTTTCC
Hu_VH_MTPX_17	CACTCTTTCCCTACACGACGCTCTTCCGATCTATGGACTGCACCTGGAGGATCCTCCTC
Hu_VH_MTPX_18	CACTCTTTCCCTACACGACGCTCTTCCGATCTTGGCTGAGCTGGGTTTYCCTTGTTGC

Hu_VH_MTPX_19	CACTCTTTCCCTACACGACGCTCTTCCGATCTGGAGTTKGGGCTGMGCTGGGTTTTCC
Hu_VH_MTPX_20	CACTCTTTCCCTACACGACGCTCTTCCGATCTGCACCTGTGGTTTTTCCTCCTGCTGGTG
Hu_VH_MTPX_21	CACTCTTTCCCTACACGACGCTCTTCCGATCTCACCTGTGGTTCTTCCTCCTSCTGG
Hu_VH_MTPX_22	CACTCTTTCCCTACACGACGCTCTTCCGATCTCCAGGATGGGGTCAACCGCCATCCTC
Hu_VH_MTPX_23	CTCTTTCCCTACACGACGCTCTTCCGATCTCAGAGGACTCACCATGGAGTTTGGGCTGAG
Hu_VH_MTPX_24	CCTACACGACGCTCTTCCGATCTGGACTCACCATGGAGTTGGGACTGAGC
Hu_VH_MTPX_25	CCTACACGACGCTCTTCCGATCTGGGCTGAGCTGGCTTTTTCTTGTGGC
Hu_VK_MTPX_1	CTACACTCTTTCCCTACACGACGCTCTTCCGATCTATGTTGCCATCACAACTCATTGGGTTTCTG
Hu_VK_MTPX_2	CTACACTCTTTCCCTACACGACGCTCTTCCGATCTATGGAARCCCCAGCGCAGCTTCTCTTCC
Hu_VK_MTPX_3	CTACACTCTTTCCCTACACGACGCTCTTCCGATCTATGAGGCTCCCTGCTCAGCTCTTGGGGCT
Hu_VK_MTPX_4	CTACACTCTTTCCCTACACGACGCTCTTCCGATCTATGAGGCTCCCTGCTCAGCTCCTGGGGCT
Hu_VK_MTPX_5	CTACACTCTTTCCCTACACGACGCTCTTCCGATCTATGGACATGAGGGTCCCTGCTCAGC
Hu_VK_MTPX_6	CTACACTCTTTCCCTACACGACGCTCTTCCGATCTATGGACATGAGRGTCCTCGCTCAGC
Hu_VK_MTPX_7	CTACACTCTTTCCCTACACGACGCTCTTCCGATCTATGGAAGCCCCAGCACAGCTTCTTCTTCC
Hu_VK_MTPX_8	CTACACTCTTTCCCTACACGACGCTCTTCCGATCTATGAGGCTCCTTGCTCAGCTTCTGGGGCT
Hu_VK_MTPX_9	CTACACTCTTTCCCTACACGACGCTCTTCCGATCTATGGAAGCCCCAGCTCAGCTTCTCTTCC
Hu_VK_MTPX_10	CTACACTCTTTCCCTACACGACGCTCTTCCGATCTATGGACATGAGGGTCCCCGCTCAGC
Hu_VK_MTPX_11	CTACACTCTTTCCCTACACGACGCTCTTCCGATCTATGGGGTCCCAGGTTCACCTCCTCAG
Hu_VK_MTPX_12	CTACACTCTTTCCCTACACGACGCTCTTCCGATCTATGGTGTTGCAGACCCAGGTCTTCATTTC
Hu_VK_MTPX_13	CTACACTCTTTCCCTACACGACGCTCTTCCGATCTATGGACATGAGGGTGCCCGCTCAGC
Hu_VK_MTPX_14	CTCTTTCCCTACACGACGCTCTTCCGATCTCAGGAAGATGTYGCCATCACAACTCATTGG
Hu_VK_MTPX_15	CACTCTTTCCCTACACGACGCTCTTCCGATCTCTCRCAATGAGGCTCCCTGCTCAGCTC
Hu_VK_MTPX_16	CACTCTTTCCCTACACGACGCTCTTCCGATCTCCTGCTCAGCTCYTGGGGCTGCTAATGC
Hu_VK_MTPX_17	CACTCTTTCCCTACACGACGCTCTTCCGATCTATGGACATGAGGGTGCCCGCTCAGCGCC
Hu_VK_MTPX_18	CACTCTTTCCCTACACGACGCTCTTCCGATCTATGGACATGAGGGTSCCYGCTCAGCKCC
Hu_VK_MTPX_19	CACTCTTTCCCTACACGACGCTCTTCCGATCTGCTCCTGGGGCTGCTAATGCTCTGG
Hu_VK_MTPX_20	CACTCTTTCCCTACACGACGCTCTTCCGATCTGGGGCTCCTGCTGCTCTGGCTCC
Hu_VK_MTPX_21	CACTCTTTCCCTACACGACGCTCTTCCGATCTGGACATGAGGGTCCCCGCTCAGCTCC
Hu_VL_MTPX_1	CTACACTCTTTCCCTACACGACGCTCTTCCGATCTATGGCCTGGGCTCCACTACTTCTCACCCTCC
Hu_VL_MTPX_2	CTACACTCTTTCCCTACACGACGCTCTTCCGATCTATGGCCTGGTCCCCTCTCTTCCTCACCCT
Hu_VL_MTPX_3	CTACACTCTTTCCCTACACGACGCTCTTCCGATCTATGGCCTGGGCTCTGCTCCTCCTCACCCT
Hu_VL_MTPX_4	CTACACTCTTTCCCTACACGACGCTCTTCCGATCTATGGCCTGGAYCCCTCTCCTGCTCCCCCTC
Hu_VL_MTPX_5	CTACACTCTTTCCCTACACGACGCTCTTCCGATCTATGGCCTGGGCTCTGCTGCTCCTCACTCT
Hu_VL_MTPX_6	CTACACTCTTTCCCTACACGACGCTCTTCCGATCTATGGCATGGATCCCTCTCTTCCTCGGCGTC
Hu_VL_MTPX_7	CTACACTCTTTCCCTACACGACGCTCTTCCGATCTATGGCATGGGCCACACTCCTGCTCCCACTC

Hu_VL_MTPX_8	CTACACTCTTTCCCTACACGACGCTCTTCCGATCTATGGCCTGGGTCTCCTTCTACCTACTGCCCT
Hu_VL_MTPX_9	CTACACTCTTTCCCTACACGACGCTCTTCCGATCTATGGCCTGGACTCCTCTTCTTCTCTTGCTCCT
Hu_VL_MTPX_10	CTACACTCTTTCCCTACACGACGCTCTTCCGATCTATGGCCTGGACTCCTCTCCTCCTCCTGYTCC
Hu_VL_MTPX_11	CTACACTCTTTCCCTACACGACGCTCTTCCGATCTATGAGTGTCCCCACCATGGCCTGGATGATGC
Hu_VL_MTPX_12	CTACACTCTTTCCCTACACGACGCTCTTCCGATCTATGGCCTGGGCTCCTCTGCTCCTCACCCTCC
Hu_VL_MTPX_13	CTACACTCTTTCCCTACACGACGCTCTTCCGATCTATGRCCDGCTTCCCTCTCCTCCTCACCCT
Hu_VL_MTPX_14	CTACACTCTTTCCCTACACGACGCTCTTCCGATCTATGGCCTGGACCCCACTCCTCCTCCTCTTCC
Hu_VL_MTPX_15	CTACACTCTTTCCCTACACGACGCTCTTCCGATCTATGGCCTGGGCTCTGCTSCTCCTCASCCT
Hu_VL_MTPX_16	CTACACTCTTTCCCTACACGACGCTCTTCCGATCTATGGCCTGGATCCCTCTACTTCTCCCCCTC
Hu_VL_MTPX_17	CTACACTCTTTCCCTACACGACGCTCTTCCGATCTATGGCCTGGACCSCTCTCCTCCTCRGCCTC
Hu_VL_MTPX_18	CTACACTCTTTCCCTACACGACGCTCTTCCGATCTATGGCCTGGACTCTTCTCCTTCTCGTGCTCC
Hu_VL_MTPX_19	CTACACTCTTTCCCTACACGACGCTCTTCCGATCTATGGCCTGGTCTCCTCTCCTCCTCACTCT
Hu_VL_MTPX_20	CTACACTCTTTCCCTACACGACGCTCTTCCGATCTATGCCCTGGGCTCTGCTCCTCCTGACCCT
Hu_VL_MTPX_21	CTACACTCTTTCCCTACACGACGCTCTTCCGATCTATGGCCTGGACCCCTCTCTGGCTCACTCTC
Hu_VL_MTPX_22	CTACACTCTTTCCCTACACGACGCTCTTCCGATCTATGGCCTGGACCGCTCTCCTTCTGAGCCTC
Hu_VL_MTPX_23	CTACACTCTTTCCCTACACGACGCTCTTCCGATCTATGGCTTGGACCCCACTCCTCTTCCTCACC
Hu_VL_MTPX_24	CTACACTCTTTCCCTACACGACGCTCTTCCGATCTATGGCCTGGACTCCTCTCTTTCTGTTCCTCC
Hu_VL_MTPX_25	CACTCTTTCCCTACACGACGCTCTTCCGATCTATGGCCTGGACTCTTCTCCTTCTCGTG
Hu_VL_MTPX_26	CACTCTTTCCCTACACGACGCTCTTCCGATCTATGGCCTGGACTCCTCTYCTYCTCYTG
Hu_VL_MTPX_27	CACTCTTTCCCTACACGACGCTCTTCCGATCTATGGCCTGGACCCCACTCCTCCTC
Hu_VL_MTPX_28	CACTCTTTCCCTACACGACGCTCTTCCGATCTATGGCCTGGGTCTCCTTCTACCTACTGC
Hu_VL_MTPX_29	CACTCTTTCCCTACACGACGCTCTTCCGATCTGCAGCATCGGAGGTGCCTCAGCCATG
Hu_VL_MTPX_30	CACTCTTTCCCTACACGACGCTCTTCCGATCTGGCAGAACTCTGGGTGTCTCACCATG
Hu_VL_MTPX_31	CACTCTTTCCCTACACGACGCTCTTCCGATCTGCAGCACTGGTGGTGCCTCAGCCATG
Hu_VL_MTPX_32	CACTCTTTCCCTACACGACGCTCTTCCGATCTGGGCTCTGCTSCTCCTCACYCTCCT
Hu_VL_MTPX_33	CACTCTTTCCCTACACGACGCTCTTCCGATCTGGGCTCTGCTCCTCCTGACCCTC

Supplementary table 3. Overview of the adapter extension PCR primers. Letters in bold indicated the index sequence, while letters underlined indicated the Illumina P5/P7 adapter sequence.

Primer name	Sequence
**5' primers**	
P5_R1	AATGATACGGCGACCACCGAGATCTACACTCTTTCCCTACACGACGCTCTTCCGATCT
**3' primers**	
P7_R2_I1	CAAGCAGAAGACGGCATACGAGAT**CGTGAT**GTGACTGGAGTTCAGACGTGTGCTCTTCCGATCT

P7_R2_I2	CAAGCAGAAGACGGCATACGAGAT**ACATCG**GTGACTGGAGTTCAGACGTGTGCTCTTCCGATCT
P7_R2_I3	CAAGCAGAAGACGGCATACGAGAT**GCCTAA**GTGACTGGAGTTCAGACGTGTGCTCTTCCGATCT
P7_R2_I4	CAAGCAGAAGACGGCATACGAGAT**TGGTCA**GTGACTGGAGTTCAGACGTGTGCTCTTCCGATCT
P7_R2_I5	CAAGCAGAAGACGGCATACGAGAT**CACTGT**GTGACTGGAGTTCAGACGTGTGCTCTTCCGATCT
P7_R2_I6	CAAGCAGAAGACGGCATACGAGAT**ATTGGC**GTGACTGGAGTTCAGACGTGTGCTCTTCCGATCT
P7_R2_I7	CAAGCAGAAGACGGCATACGAGAT**GATCTG**GTGACTGGAGTTCAGACGTGTGCTCTTCCGATCT
P7_R2_I8	CAAGCAGAAGACGGCATACGAGAT**TCAAGT**GTGACTGGAGTTCAGACGTGTGCTCTTCCGATCT
P7_R2_I9	CAAGCAGAAGACGGCATACGAGAT**CTGATC**GTGACTGGAGTTCAGACGTGTGCTCTTCCGATCT
P7_R2_I10	CAAGCAGAAGACGGCATACGAGAT**AAGCTA**GTGACTGGAGTTCAGACGTGTGCTCTTCCGATCT
P7_R2_I11	CAAGCAGAAGACGGCATACGAGAT**GTAGCC**GTGACTGGAGTTCAGACGTGTGCTCTTCCGATCT
P7_R2_I12	CAAGCAGAAGACGGCATACGAGAT**TACAAG**GTGACTGGAGTTCAGACGTGTGCTCTTCCGATCT

Supplementary table 4. Antibodies and reagents used for flow cytometry and cell hashing.

**Item**	**Supplier**	**Product code**
Human Fc Blocker	BD	564220
anti-CD45-AF488	Biolegend	304019
anti-CD3-AF700	Biolegend	317339
anti-CD19-BV421	Biolegend	302233
anti-CD20-PE/Cy7	Biolegend	302311
anti-CD38-PE/Dazzle	Biolegend	356629
7-AAD	Miltenyi Biotec	130-120-640
TotalSeq-C0251 anti-human Hashtag 1	Biolegend	394661
TotalSeq-C0252 anti-human Hashtag 2	Biolegend	394663

Supplementary file 1: Recovered VDJ sequences from Ab-seq peptides (link)
